# Tactowel: A Subtle Sports Performance Display for Giving Real-Time Performance Feedback in Tennis

**DOI:** 10.3390/s21134594

**Published:** 2021-07-05

**Authors:** Hayati Havlucu, Aykut Coşkun, Oğuzhan Özcan

**Affiliations:** Arçelik Research Center for Creative Industries, Koç University, Sariyer, Istanbul 34450, Turkey; aykutcoskun@ku.edu.tr (A.C.); oozcan@ku.edu.tr (O.Ö.)

**Keywords:** subtle displays, ambient devices, tangible interaction, sports feedback, tennis

## Abstract

Sports technology enhances athletes’ performance by providing feedback. However, interaction techniques of current devices may overwhelm athletes with excessive information or distract them from their performance. Despite previous research, design knowledge on how to interact with these devices to prevent such occasions are scarce. To address this gap, we introduce subtle displays as real-time sports performance feedback output devices that unobtrusively present low-resolution information. In this paper, we conceptualize and apply subtle displays to tennis by designing Tactowel, a texture changing sports towel. We evaluate Tactowel through a remote user study with 8 professional tennis players, in which they experience, compare and discuss Tactowel. Our results suggest subtle displays could prevent overwhelming and distracting athletes through three distinct design strategies: (1) Restricting the use excluding duration of performance, (2) using the available routines and interactions, and (3) giving an overall abstraction through tangible interaction. We discuss these results to present design implications and future considerations for designing subtle displays.

## 1. Introduction

Sports performance feedback is crucial for athletes to enhance their performance, increase their self-efficacy and motivation [1,2]. Currently, sports technologies and wearable devices aid athletes in providing various physiological and physical metrics on their performance (i.e., heart rate) [3]. However, previous research reveals that sportspeople find the information shared by these devices as irrelevant and not valuable [4]. Consequentially, majority of the sportspeople abandon these devices within the first six months, despite the potential benefits [5,6].

Sports research prototypes and commercially available devices (i.e., mobile applications, activity trackers, and wearable devices) usually target the self-paced individual sports, like running and cycling [7,8]. Yet, there are also externally-paced individual sports, such as tennis, that are played directly against an opponent and have different requirements [9,10]. Previous research shows that tennis players expect real-time sports performance feedback (i.e., “Your head is not in the game”) from these technologies, instead of physiological metrics provided by them (i.e., burnt calories) [11]. Tournaments, such as 2019 Next Gen ATP Finals, introduced use of wearable devices in professional tennis for the first time, further endorsing this need. Unfortunately, no information about the specific performance metrics or procedures is publicly available, except that the allowed device was Catapult, a chest worn band that primarily measures hear rate.

On the other hand, providing such performance metrics in real-time is troublesome [1,7,12,13]. According to Cognitive Load Theory [14] and Applied Attention Theory [15], the interaction techniques of the current devices may overwhelm tennis players with excessive information or distract them from the game, while they are playing. Yet, previous research rarely addresses how to design feedback to prevent overwhelm and distraction [7]. Despite the work on ambient displays utilizing peripheral attention [16] and wearable devices abstracting performance [17], players’ performances are interrupted by having the feedback available all the time [18]. Thus, designing real-time sports performance feedback requires in depth explorations to prevent overwhelming and distracting tennis players.

In this paper, we propose *subtle displays* that are aimed at unobtrusively displaying low-resolution real-time sports performance feedback to prevent overwhelming and distracting athletes. Unlike ambient, glancable, and peripheral displays, *subtle displays* withhold their interactive qualities during athletes’ performance and use focused attention of tangible interaction to display feedback instead of peripheral attention [19,20]. Specifically, *subtle displays* are ambient devices in the form of augmented sports objects that display an overall abstraction of sports performance through tangible interaction only when athletes have explicit access. We apply and conceptualize *subtle displays* to tennis by designing *Tactowel*. *Tactowel* is an augmented sports towel that subtly displays low-resolution tennis performance representations by changing its texture and can be unobtrusively accessed only during the breaks. We discuss our design rationale for *Tactowel* and *subtle displays* through three distinct design strategies: (1) Restricting the use excluding duration of performance, (2) using the available routines and interactions, and (3) giving an overall abstraction through tangible interaction.

We evaluated *Tactowel* through a user study with 8 professional tennis players. In this user study, participants compared *Tactowel* with an upper end, commercially available Apple Watch application, *TennisKeeper*, to draw a baseline. Although Sony Smart Tennis Sensor and Catapult are widely used in Tennis, TennisKeeper was chosen because it shared the most amount of tennis related performance metrics (i.e., shot angles and percentages) with the players, through an accustomed visual display in real-time. First, the players experienced both modalities remotely through video-sketches [21]. Then, they filled a 5-point Likert scale questionnaire that contained our design claims, which is an established methodology for evaluating design decisions and strategies [22]. This questionnaire was utilized to prime participants to think about these claims in the following focus group interviews and not to quantify the results. The result of these focus group interviews indicate that *subtle displays* could prevent overwhelming and distracting tennis players through our three design strategies. We further discuss these results to present design implications and future considerations for designing *subtle displays*.

We argue our work contributes to the design knowledge on how to design feedback modalities and interactions for sports technologies by introducing the novel concept of *subtle displays* and implementing its theoretical knowledge to *Tactowel* by three distinct design strategies. These design strategies and their design implications can guide feedback designers, human-computer interaction, and sports researchers to design unique feedback modalities and interactions for high performance activities that require real-time performance feedback without interruption.

## 2. Background & Related Work

Sports feedback is the information presented to athletes on their behavior and performance [23,24]. Previous research exhibits receiving sports feedback is critical for sportspeople’s development, as well as to increase their motivation and self-efficacy [1,2]. To receive this feedback, athletes who do not have access to trainers or coaches use sports technologies, especially wearable devices, which provide various physiological metrics on the performance, such as heart rate or burnt calories [3]. Regardless of the potential advantages, studies show every third of wearable device users abandons these devices in the first six months [5,6]. According to Reference [4], the main cause of the high abandonment rates is the presented data. This research emphasizes sportspeople do not gain any new, useful, or valuable information from these devices. In addition, this information remains unprocessed, thus not being actionable for many sportspeople.

### 2.1. Tennis and Externally-Paced Sports

Most commonly, sports technologies are targeted at sports, like running [7], swimming [17], and cycling [8]. These sports only involves self-paced performances, meaning the athletes decide and initiate their performance without any interference from other athletes [25]. Thus, the technologies largely present data on the outcome of the athletes’ performance (i.e., the distance, speed, steps) [24,26]. However, sports, like tennis, generally involve externally-paced performances, in which the athletes react to the direct actions of the opponent [10]. This contextual difference creates different user needs and requirements, which are not fulfilled by current sport technologies [4]. For example, tennis players need to pay attention to the minimal cues to anticipate the opponent’s actions, quickly decide on an counter-action and rapidly perform it [9]. Therefore, the players’ overall speed or burnt calories remain irrelevant to their actual performance. In this direction, ref. [11] has shown that tennis players need to have feedback on the characteristics of their performance (mental states and behavior) as their coaches give, while they are performing. On the other hand, providing many performance metrics in real-time is found to be challenging because this could overwhelm the athletes with excessive information and distract them from their performance [1,7,12,13].

### 2.2. Cognitive Load Theory

According to Cognitive Load Theory (CLT), capacity of the working memory, which processes the received information in real-time, is limited [14]. Athletes already receive and process vast amount of intrinsic information during the intensity of the game play [9]. Thus, they push the limits of their working memory. Augmented (additional) information exerts extra loads to this capacity. If the amount of augmented information surpasses the limits of working memory’s capacity, it causes a cognitive overload [27]. In return, athletes feel overwhelmed by the redundant information and are rendered unable to take action. Previous studies demonstrate the effects of CLT. For instance, studies on wearable devices shows presenting high amount of data during exercise can increase the cognitive load on athletes [28]. In this direction, abstracting the data and reducing the amount of information is found to decrease the cognitive load [12,29]. Work on ambient displays, uses similar principles to abstract the information and report lower cognitive load. Thus, reducing the amount and resolution of information was also found less overwhelming [30]. Still, these principles are not well integrated into current sports technologies that present feedback in real-time [12].

### 2.3. Applied Attention Theory

Applied Attention Theory (AAT) acknowledges the finite attentional resources in divided attention situations, such as performing a sport [15]. It suggests augmented information diverts the attention of the athletes away from their performance [31]. However, athletes need to focus their full attention on the performance [9,10]. Previous research demonstrates receiving information in these contexts intrudes the performance [26]. Several researches support AAT’s claims. For example, notifications present feedback in divided-attention situations [31]. They claim the interruption level of the feedback increases when athletes need a better comprehension of the feedback. Work on real-time feedback illustrates augmented information attracts an external focus of attention and divides the focused attention on sports performance [26]. Moreover, it suggests this feedback intrudes the performance of the athletes and are found annoying [32]. Studies on wearable devices demonstrate just having a device available can prevent athletes from concentrating on their performance [29]. These situations of divided attention cause athletes to get distracted.

Overall, despite the demand for receiving real-time sports performance feedback, current feedback modalities can overwhelm and distract the athletes. Addressing these issues, wearable devices use abstractions to decrease the cognitive load of the feedback [17]. Ambient displays use periphery of athletes’ attention to decrease the attention demand [16]. Nonetheless, athletes could access the feedback from these devices in the time of need, which is found to distract the athletes [18]. In this direction, former studies also present the techniques of different feedback modalities (see Reference [12] for a detailed review). Yet, they also note that these modalities cannot be compared without assessing the design and the context of the feedback. Few research studies address how to design feedback to prevent overwhelm and distraction [7]. In this regard, designing real-time sports performance feedback that can satisfy the needs of externally-paced sportspeople, such as boxers, fencers, any form of martial artists, and racket sportspeople, including tennis players, requires further investigation to prevent overwhelming and distracting these athletes. We propose subtle displays to address this gap. The following section elaborates on subtle displays and situates these in relation to related work.

## 3. Subtle Displays

Subtle, an adjective, defines something that is few but critical, which is accomplished by not demanding too much attention. We propose subtle displays as real-time sports performance feedback output devices that unobtrusively present low-resolution information. Low-resolution is a term used for displays that presents information that is usually not well-defined. Nonetheless, a low-resolution output helps to perceive ‘the bigger picture’ without getting lost in the details. Subtle displays are both inspired from elite tennis coaches’ approaches to give real-time sports performance feedback and a merger between ambient and tangible displays [20,33,34].

Previous work strongly argues that designers must learn and apply coaches’ techniques to sports technologies [7,35,36]. Havlucu et al. interviewed 6 elite tennis coaches to learn their approaches for giving real-time sports performance feedback [33]. Their findings indicate that elite coaches refrain from intervening the players and remain passive until the players either demand the feedback or require assistance. When they give feedback, the coaches only share an overall abstraction of the performance (i.e., “You are doing great”) to raise the awareness of their players and expect them to find their own solutions. According to their discussion, a real-time sports performance feedback should give low-resolution information not to overwhelm the players and should be unobtrusively given when the players need the feedback not to demand too much attention. Similar to coaches approaches, ambient displays give low-resolution information using abstractions, such as color changes or sounds [37]. This is found to be decreasing the cognitive load of the feedback; thus, it could prevent athletes from feeling overwhelmed [12,20,37].

However, previous studies show ambient displays could become obtrusive [29,33]. Ambient and peripheral displays use periphery of the users’ attention and users’ decision to receive feedback to prevent distraction from primary task [16]. Athletes are distracted by being able to access the performance feedback, when they feel stressed about their performance [18,38]. Thus, these displays should avoid giving feedback to athletes in attention demanding phases, like elite tennis coaches who avoid their players by averting their gaze [33]. In this regard, we argue real-time sports performance displays should not use the periphery of athletes’ attention. Instead, they should be peripheral or ambient devices that wait for athletes to demand the feedback and could use focused interaction to display the feedback. A possible way to achieve this is to restrict the time intervals these devices could be accessed. If these devices can only be remembered and can only display feedback during certain available times, athletes would not be distracted by seeking information. Thus, we speculate subtle displays as ambient devices that withhold their interactive qualities during athletes’ performance. They use focused interaction to display feedback, only when the athlete can spare their attention.

In addition, ambient displays commonly use visual and auditory modalities to capture the peripheral attention of the users [16,37]. However, like most sports, tennis is cognitively mastered according to the visual and auditory anticipation (i.e., seeing or hearing the ball to decide on the next move) [12,39]. According to modality effect theory, the working memory can only process one type of modality at a given time, either the feedback or the anticipatory cue [14]. Therefore, it suggests that using other modalities, such as haptic feedback and tangible interaction, could prevent overwhelming and distracting athletes [12,14,39]. Research on ambient and peripheral displays were also curious how would tangible interaction could be integrated to these displays [40]. Tangible displays could be beneficial because they use the physicality of everyday objects and human skills to display information, rather than relying on cognitive skills and digital representations [34]. This allows tangible displays to imitate the usage context of sports objects to convey sports performance feedback naturally, which can also prevent overwhelming and distracting athletes [19]. Therefore, subtle displays are proposed as ambient displays that use the focused attention of tangible interaction instead of peripheral attention. They are augmented sports objects that are found in the ambience of sport environment, which use the natural interaction principles of tangible interaction to display real-time sports performance feedback. They utilize the usage contexts and physicality of these objects as interactive qualities.

To sum up, subtle displays are ambient devices in the form of augmented sports objects that display an overall abstraction of sports performance through tangible interaction only when athletes have explicit access. Unlike ambient, glancable, and peripheral displays, subtle displays withhold their interactive qualities during athletes’ performance and use their focused attention instead of peripheral attention. They are aimed at unobtrusively displaying low-resolution real-time sports performance feedback to prevent overwhelming and distracting athletes. Although there are many different sports technology applications and research, we did not come across a subtle real-time sports performance display that intends to prevent overwhelming and distracting tennis players. The closest example of a subtle display is presented by Hudson and Harrison outside the scope of real-time sports performance feedback [41]. They developed texture displays for attention demanding situations, such as a business meeting. Texture displays change the qualities of their texture to passively display state information. The authors argue these displays would not distract their users because they give the active role to the user to inquire the feedback and do not alert with an active output (i.e., vibrotactile feedback for incoming message).

### 3.1. Tactowel

**Figure 1:**
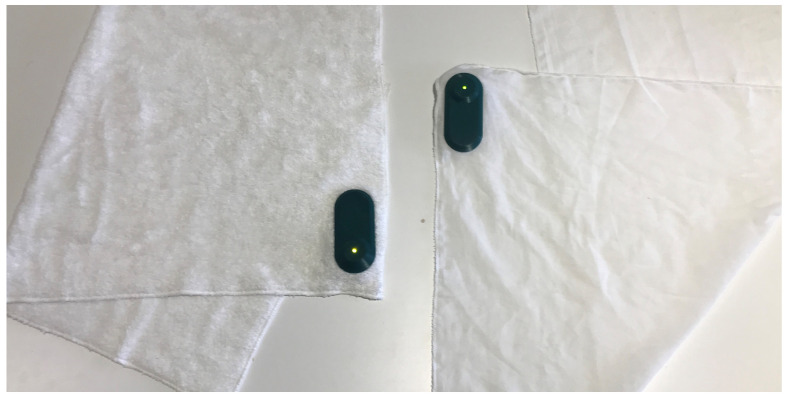
The conceptual prototype of Tactowel. Soft and hard textures are represented by two different towels. The soft Tactowel (**left**) is 40% bamboo-60% cotton mix bath towel and the hard Tactowel (**right**) is 100% cotton peshtemal. The green hard case is a dummy device that contains LED for participants to believe it is fully-functioning.

We designed Tactowel to conceptualize a subtle display that could give real-time sports performance feedback in tennis. Inspired by texture displays, Tactowel is an augmented sports towel that subtly displays low-resolution sports performance representations by changing its texture (Figure 1). It can be unobtrusively accessed only during the breaks. We elaborate on our design rationale and strategies in what follows.

#### 3.1.1. Restricting the Use of Tactowel Excluding the Duration of the Performance

It should only be used when players are not performing to prevent distracting them from their performance. However, it should also be used during the tennis match. We identified that tennis has frequent breaks between every point, game and set. These breaks could create time intervals for communicating sports performance feedback, without interrupting the actual performance. Other externally-paced sports, such as fencing, boxing, and wrestling, also have these breaks. With this potential, we decided that Tactowel should only be accessed during breaks.

#### 3.1.2. Using the Available Routines and Interactions of Tactowel to Display Sports Performance Feedback

It should be an augmented sport object that is found in the environment and should be used naturally as that object is used in its context. However, it should not be a conventional sports equipment that could be accessed by the players during their performance (i.e., tennis racket). In this direction, ref. [42] designed and discussed multiple concepts for giving feedback in tennis. Their results suggest tennis players’ rituals, routinized behavior, could be observed an utilized to design a modality. For this reason, we observed what tennis players do during the breaks and discovered that tennis players frequently used their towels. The players touch, bend, grip, and do many other actions with their towel, which could create ways to display sports performance feedback using the natural interaction with the towel. Thus, we decided Tactowel should be an augmented sport towel.

#### 3.1.3. Giving an Overall Abstraction of Sports Performance through Tactowel’s Tangible Interaction

It should represent the information elite coaches share (i.e., “You are doing great” or “You can do better”), which could also be explained as performance states (i.e., good performance or bad performance), to prevent overwhelming the players. Additionally, the use of tangible interaction ensures that players could not perceive Tactowel displaying feedback when they are performing and would not get distracted by it. Tangible interaction uses physicality of the objects as interactive qualities [19,34]. In this regard, ref. [43] specified textural properties of a sports towel according to professional athletes’ association with their performance. For this reason, we decided to use the texture of Tactowel to display different performance states. Different textural states of Tactowel, display different performance states (i.e., soft-good performance, hard-bad performance). When players touch Tactowel, they receive the sports performance feedback.

## 4. Methodology

In this paper, we evaluate our approach to subtle displays and Tactowel with a user study. Our aim in this study is to explore subtle displays and our design strategies implemented in Tactowel for giving real-time sports performance feedback without overwhelming and distracting tennis players.

McCrickard et al. argues designers can evaluate their prototypes and design decisions through claims [22]. In Scope’s project, they present a claim-specific questionnaire through which they evaluated the success of their design strategies. Due to our coincided goal, we use the same approach to create our own questionnaire items that communicate our design goals to the participants and allow them to rate their agreement according to their experience with the modality (Table 1). Each item refers to a feedback quality procured from the literature (see Reference [12]) but does not specify the design decision or the modality (i.e., 17. Distraction—With this modality, the performance feedback would not distract me from my performance.). Only one item, cognitive load/overwhelm, is procured from Mental Effort Rating Scale [14], as this item was a pre-established method for measuring cognitive load. Each item was written in English and back-translated to Turkish twice, by three external researchers [44]. Both versions of the items were sent to a previously professional tennis player. This player was asked to briefly explain what he understood from each item and the researcher evaluated how well each item communicates the intended goal and iterated accordingly. We should emphasize that this questionnaire is aimed to explore the design strategies, not to validate them. We utilized this questionnaire to prime the participants to think about these feedback qualities and help them reflect on these during focus group interviews and not to quantify any results.

### 4.1. Procedure

**Figure 2:**
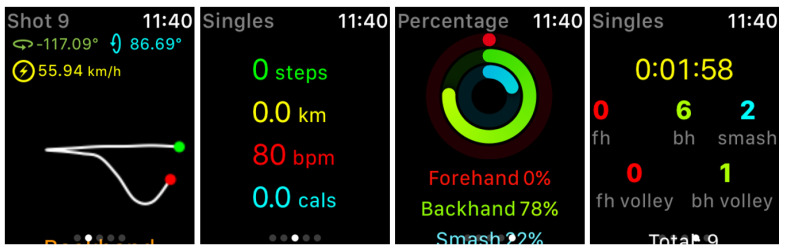
Screenshots from TennisKeeper showing (1) shot angle and speed, (2) steps, distance, hearth-rate and calories, (3) percentage of shot types, and (4) quantity of shot types.

The study was conducted via Zoom, during COVID-19 pandemic (see Section 7). The researcher first introduced and demonstrated the two modalities including Tactowel (Figure 1), starting from TennisKeeper. TennisKeeper is an application for Apple Watch that allows players to visually access a variety of detailed performance metrics in real-time (Figure 2). This modality is chosen because it reflects the upper end of available performance displays with high amount and resolution of visual information. Thus, it can help to set a baseline for evaluating the design decisions implemented in Tactowel and help to learn if certain feedback qualities are lost in the process. However, we should note that there are a variety of devices and modalities for tennis, the most dominant being Sony Smart Tennis Sensor, a device that is attached on the bottom of the racket and Catapult, a wearable chest band. Yet, such devices are left out in this study because both Sony Smart Tennis Sensor and Catapult only measure specific performance metrics (i.e., shot angle and heart rate respectively) and use a mobile phone application to share these, which prevent athletes to receive real-time feedback due to limited access to their phones during games.

Then, the researcher sent a link to an online survey that included the 5-point Likert scale questionnaire to prime the participants to think about the feedback qualities of both modalities (Table 1) and their video-sketches [21], which contextualized the modalities’ use in tennis and helped participants to experience them remotely. Participants were asked to watch the video-sketches first and ask questions if they are confused, and then to fill out the questionnaire for each modality. The order of both modalities was randomized to prevent bias. After experiencing the modalities remotely, focus group interviews were conducted. Participants were asked to bring a regular towel and a watch to the interviews to help them contextualize their experience. During the interviews, they were encouraged to think with these objects to imagine the modalities in the discussed situation. Yet, there were only few instances where this was observed. Each focus group interview lasted around 45 min and video recorded. Later, the recordings were transcribed and coded by the researcher according to the codes presented in Table 1. These codes were then analyzed by content analysis to produce the themes presented in Section 5 [45].

### 4.2. Participants

Eight professional tennis players participated to this study. We should emphasize that finding eligible participants on this level is extremely challenging as also presented by References [11,33,42]. In addition, previous research demonstrates more than 7 participants are optimal for small scale user testing [46]. However, research examples including elite and professional level participants have been published with as low as 3 participants [47]. We contacted the participants through Turkish Tennis Federation; thus, all participants are from Turkey. Although the participants are young (M = 18.38, SD = 4.03, in years) (5 females), they are highly experienced in tennis (M = 11.50, SD = 3.37, in years). They train every day and all of them have won tournaments on national and international levels. All participants shared that they can easily lose track of their performance during these tournament games and they wish to receive performance feedback during games. 6 participants track their performance by receiving feedback from their coach. However, only 3 participants used wearable devices before to track their performance, including Apple Watch and Polar, only for a short period of time. Participants think that a good tennis performance is when they stay in the game and play flawlessly, independently from winning or losing, and their mental states are crucial to achieve this. Thus, they want to receive mental and motivational information from these devices, together with their winning moves and mistakes.

## 5. Results

### 5.1. Questionnaire

The difference between the ratings of the questionnaire items for TennisKeeper and Tactowel was analyzed by Repeated Measures *t*-test (Table 2). The results of the analysis show that there is no significant difference between both modalities for displaying real time performance feedback. However, note that these items were rated prior to the focus group interviews, and the aim was not to quantify the results, but to prime the participants to reflect on these feedback qualities. Thus, we observed that the participants ideas about the modalities change over the focus group interviews and these results only represent their initial thoughts.

Most items were rated favoring TennisKeeper. This was expected due to some players previously using similar modalities, such as Apple Watch, and the effect of legacy bias, that comes from prior experience, on participant ratings [48]. The highest and most significant differences were observed for relevance (3) and discovery (8). Overall, participants thought that the variety of information shared by TennisKeeper is more relevant to tennis than Tactowel’s abstraction and this would allow them to learn something new about their performance. Yet, ratings of resolution (3), perception (10), and distraction (17) favored Tactowel. Participants thought the information from TennisKeeper is too detailed. This would make the feedback to be perceived harder and distract the players from their performance compared to Tactowel. Therefore, an initial insight is that lowering the detail of the information as it was applied to Tactowel could make the feedback easily perceived and prevent distraction. Surprisingly, participants rated motivation (18) and companion (20) equally for both modalities. This means that any form of feedback could support the players and increase their motivation during the game. Thus, it highlights the need of receiving feedback during games.

The following sections elaborate the dominant themes from the focus group interviews according to our content analysis [45].

### 5.2. Amount and Resolution of Performance Feedback during the Game

P2—*“This [TennisKeeper] is the coach. This [Tactowel] is nice… Comfortable. The less information is better… The coach talks a lot and irritates me.”*

Overall, the participants thought having more detailed information is important to increase their awareness on their performance during the game. Four participants mentioned that they want to receive detailed information, such as their shot percentages or speed, because it could make a difference. They thought lowering the resolution of the information would make the feedback unclear. Thus, it would be harder for them to understand. On the other hand, 5 participants highlighted that detailed information is not always helpful because most of the information, such as the number of forehands or backhands, is not relevant during their performance. They were fed up with receiving a lot of information on their tennis performance during games as these could overwhelm them. For this reason, they liked Tactowel better and found TennisKeeper annoying. These players addressed that low-resolution information, like their performance states, could still increase their awareness to some extent. They implied that they could discover their shortcomings without the detailed tactical information and create their own solutions. Additionally, all participants found detailed information is not suitable for during the game, but after the game. They thought Tactowel would give an overall sense of awareness during the game and TennisKeeper would present the solutions after the game or during practice. For this, they suggested to combine these displays.

### 5.3. Feeling Overwhelmed by Receiving Performance Feedback during the Game

P7—*“I think It [TennisKeeper] could overwhelm. Sometimes the player gets irritated when a lot of things said one after another. Like ’Take It easy!’, because the player thinks what to change what to do. It touches your nerve, when you receive continuous instructions on top of that. Like ’Ok, shut up!’… Thus, you need to find its balance."*

All participants emphasized that receiving a lot of information during the game would overwhelm them. They pointed out that they could easily get confused and irritated because they are already stressed and overthinking about their performance. For this, they found TennisKeeper to be more overwhelming. In addition, the players mentioned that receiving feedback all the time would be more overwhelming. Thus, they wanted to receive performance feedback only when they need it. In this regard, 4 participants liked accessing performance feedback only during the breaks. They shared that this would also allow them more time to figure out what to do next. Therefore, they indicated that Tactowel would not overwhelm them.

### 5.4. Distraction Caused by Receiving Performance Feedback during the Game

P2—*“[TennisKeeper is more distracting]. Because it is here [holds her wrist with her hand] and will catch your eye like this [imitates striking a shot and moves her wrist in front of her eyes]. I strike a forehand like this and it goes out… I will look like this [glances at her wrist] and get distracted. However, that [Tactowel] will be there [shows the side]. I will look right after.”*

All participants highlighted that receiving detailed information during the game would distract them. They thought Tactowel would not distract them because it only gives information in low-resolution and does not focus on any detail to confuse them. In addition, the players thought being able to perceive the feedback all the time would be more distracting. Three participants emphasized that the feedback would catch their eye and intrude their performance. They suggested to place TennisKeeper out of their eyesight to prevent distraction. Thus, they liked restricting the access to the feedback and found Tactowel to be less distracting. Moreover, 3 participants indicated that perceiving performance feedback instantly could decrease distraction. For this, they shared that these displays should be less complex. Four participants also mentioned that they could get distracted if they are not accustomed to the performance display and it causes discomfort.

### 5.5. Coherence of Performance Feedback with Tennis

P8—*“I do not think [Tactowel] would overwhelm… I see towel as a place to catch breath and rest… Tennis players’ aim to going to their towels is already to catch their breath, think about their strategy in the game, to continue the same plan if everything is going well, if not in that instance… I mean, in fact just touching the towel is relaxing, but no one is aware of that.”*

The players were concerned about the coherence of using the feedback modalities with their tennis performance. Seven participants found Tactowel highly coherent because, they already utilize using their towels as a routine to reflect on their performance during the games. The players thought having the feedback in such a natural way would help to prevent overwhelming them. In addition, they mentioned that this routine would allow more time to think about their performance, compared to the instant feedback from TennisKeeper. Moreover, all participants found using Tactowel practical because the towel is already a part of tennis and they are accustomed to using it during their performance. The participants implied receiving feedback from accustomed things would not intrude their tennis performance. On the other hand, 5 of the participants suggested that visual feedback could clash with the nature of tennis. Although looking at TennisKeeper right after the point is practically similar to touching Tactowel, the players felt uncomfortable and found it unnatural.

### 5.6. Tangible Performance Feedback during the Game

P1—*“I mean it [Tactowel] will sooth you. I will look at that [the watch]. Hmm. Swipe this right. Hmm. How well I shot…I mean tell me what I need to do. Tell me the solutions. But this [Tactowel] is soft. It sooths.”*

Players emphasized playing comfortably is crucial for tennis due to its intense load of stress. In this regard, 4 participants indicated that touching different textures to receive feedback would wake different feelings to make them comfortable. They shared a soft texture when they are performing well would be soothing to affirm their good performance and a hard texture would be stimulating to push them change their bad performance. Considering this, we did not specify what kind of feedback Tactowel was giving with the texture change. We only told that it communicates the players are performing good or bad. However, 4 participants associated the textures with motivational feedback. They implied that the soothing nature of the soft texture would help them stay in the game and the stimulating nature of the hard texture would push them to win. In comparison, the players associated receiving a lot of information visually through TennisKeeper with their coach talking a lot. They mentioned this could irritate and overwhelm them during the game. In this regard, 2 participants highlighted that visual feedback would be distracting because it can catch their eye at a wrong time. They suggested the displays should be out of their eyesight. Thus, 4 participants shared receiving performance feedback through textures is advantageous because it remains hidden from their opponents and others on the court. They also found this similar to their coaches encrypted signaling during their games. Additionally, 4 participants found textures to be more memorable and easier to perceive in an instant and emphasized that these are important factors to reduce distraction.

### 5.7. Increase in Motivation by Receiving Performance Feedback during the Game

P4—*“Feeling the towel [Tactowel] could be more important for me. It is like a coach’s support from outside of the court. To feel it… I mean to have that connection is important for me… I mean something is supporting you whatever happens, still wants you to win, it does not get mad at you or it understands you. To be understood… For instance, after I lose a point and I go the towel and remind myself, ’Ok, there is nothing wrong, the next point!’.”*

Six participants highlighted the importance motivation during tennis games. They believe receiving motivational feedback is more helpful compared to receiving tactical information, such as shot percentages, while performing. In this regard, they associated the performance feedback from Tactowel to be motivational and emphasized that it could increase their motivation during the game. Four participants also stated that they would treat Tactowel as a companion, through which they would get support during the game. From time to time, we observed these participants talk about Tactowel as “we” and joke around with it to demonstrate the bond. Two participants shared that they would set a goal to keep Tactowel always soft to increase their motivation. They suggested that they will try to satisfy and please it, like a parent in the audience. However, they mentioned this could distract them because they would be always reminded of the performance feedback. In this direction, 6 participants recommended that the feedback should not communicate any negative information as it could demotivate them. However, the players were not convinced that it would be helpful without negative feedback. Instead, 4 participants thought that they could change negative feedback to be more motivational and negate its negative impact. For example, they shared that they would accept the negative feedback as a challenge between them and Tactowel to push their performance to the limits.

## 6. Discussion

Our aim is to evaluate Tactowel to inform on subtle displays. The results endorsed our problem statement and design rationale, while extending the current approach. In this section, we discuss these results according to the three distinct design strategies we implemented in Tactowel and their design implications related to subtle displays. Note that some features of these strategies are interrelated.

### 6.1. Restricting the Use of Sports Performance Displays Excluding the Duration of Performance

Subtle displays should only be used when players are not performing, but still during the game, to prevent distracting them from their performance. For this, Tactowel displays the sports performance feedback only during the breaks and restricts its use otherwise.

The interviews revealed that this was an alternative strategy to utilizing peripheral attention [20]. In line with previous work, the players shared that they would be distracted by having the feedback available at their glance as it would catch their eye and intrude their performance [18]. Although TennisKeeper closes its screen until the wrist is turned upwards, the players mentioned that they would be reminded of the possible feedback when they are in need and would be prompted to check it. On the other hand, they highlighted that Tactowel would be on the bench and out of their eyesight until the breaks. Having said that the players also claimed that receiving feedback all the time would be overwhelming. They only wanted to receive performance feedback when they need it, contradicting the continuous feedback strategy of ambient and peripheral displays [16]. Thus, they liked Tactowel’s strategy of restricting the feedback to breaks as this gives them the agency to inquire the feedback or not [41]. This suggests that restricting the time of use could be used to prevent being overwhelmed, as well as being distracted.

#### 6.1.1. Other Restricting Strategies Could Be Used, Combined and Separated

We learned that there can be multiple strategies and benefits of restricting the use of these displays. The players explained that if they are not accustomed to the display, the discomfort would also be an intruding reminder. Contrary to wearable devices, they suggested the display could be placed out of their body and out of their reach to prevent distraction, even if it is available during the game [18]. Therefore, restricting the reach to the display could be another valuable strategy. We realized that using a towel as a display consequentially restricted the reach to the display along with our initial decision of restricting the time of use. However, these restricting strategies could be separated, or different strategies could be formed in the future subtle displays. For instance, as players suggested, Tactowel could be an arm band. Since the players would need one hand touching the other arm to perceive the texture and they need one hand to hold the racket while they are playing, this would restrict the time of its use excluding the performance. However, it would not restrict the reach because it is on the body. Thus, the players can use it outside of the breaks (i.e., while the opponent is preparing to serve).

#### 6.1.2. Restriction Could Create an Additional Value of Support

The players associated Tactowel with a companion, for example as a coach or a friend at the sides, and referred to Tactowel as they were a team. They shared that they would run to Tactowel as soon as there is a break and feel its support. We believe this is due to the restricted access to the display, as the players cannot communicate with their companions during the game. However, this situation also causes a risk to further distract the players. The players claimed that they would set a goal to keep Tactowel always soft and try to satisfy it, so that it would always give positive feedback and support. Contrarily, they claimed that they would be demoralized if they would receive a negative feedback. Thus, the players could seek what Tactowel is ‘thinking’ about their performance just before the break and get distracted. In this regard, we believe the future subtle displays should find a way to support the players, even when they are displaying a negative feedback, or they should create other restricting strategies that could block the player from trying to satisfy these displays. For example, 3 participants mentioned that they would only seek feedback when they are losing because they do not want to change their winning performances. Thus, limiting the use of display according to the score could be a different restriction strategy to achieve that.

### 6.2. Using the Available Routines and Interactions to Display Sports Performance Feedback

Subtle displays should be augmented objects that are found in the environment and should be used naturally as those objects are used in their contexts to prevent distracting the players by interrupting their routine. Embodying this, Tactowel is a sports towel that displays sports performance feedback during towel routines.

The interviews affirmed that the players are concerned to interrupt their routines while playing tennis. They thought visually receiving feedback is disruptive to their tennis performance and emphasized that they cannot look at such feedback all the time as it felt unnatural [14]. Additionally, they found receiving information outside of their routine very complex and possibly distracting. In contrast, the players found receiving feedback from Tactowel by touching it very natural and practical [36]. They stressed that this interaction is already a part of their routine to catch their breath and rest. Thus, they suggested this interaction does not require any additional attention or effort and would not overwhelm them.

#### Other Routines and Interactions Could Be Used to Attribute Different Meanings to the Feedback

The players thought interaction with Tactowel was coherent with receiving the feedback because they utilize going to their towels to reflect on their performance and pull themselves together. This created an opportunity for Tactowel to support the players with motivational feedback. In this regard, we believe not only restricting the use of feedback but engaging players with the feedback in a meaningful way could prevent distracting and overwhelming them. Considering the coherence between going to a towel and motivational support, we believe there could be other routines and interactions to attribute different meanings to the feedback. Therefore, we argue that future subtle displays should explore the coherence between different routines and their meaning for giving different kinds of feedback. We believe observations are crucial to achieve this. For instance, along with the towel, we observed that during the breaks many tennis players scratch and look at their rackets’ strings to concentrate on their game and not to get distracted by the audience. Utilizing this routine, we can speculate a subtle display that vibrates these strings when they are scratched to focus the players on their game plan. Future research could observe similar routines, produce quick prototypes, such as Wizard-of-Oz [49], and evaluate the meaning of the feedback through a user study similar to the one presented in this paper.

### 6.3. Giving an Overall Abstraction of Sports Performance through Tangible Interaction

Subtle displays should give an overall abstraction of sports performance to prevent overwhelming the players and display the feedback through tangible interaction to prevent distracting them. For this, Tactowel utilizes soft and hard textures to abstract sports performance.

The interviews supported our problem statement that receiving high amount and resolution of information during the game could overwhelm the players. In line with previous work, the players pointed out that they cannot value or make use of high amounts of information during the game [18]. Instead, they feel irritated and annoyed by this situation. On the other hand, we learned that players mostly value the motivational support and can act on motivational feedback, which is to their highest benefit during the game. Similar to the work on ambient displays, the players shared that this motivational support could be achieved by an overall abstraction of their performance [16]. They emphasized this kind of abstraction can increase their awareness of their performance and they are already proficient enough to find their own solutions confirming the discovery learning theory [36].

#### 6.3.1. Overall Abstraction Could Make the Feedback Unclear

The players still wanted Tactowel to share more information because they thought lowering the resolution of the information could make the feedback unclear. They believed high-resolution feedback would increase their awareness robustly and it would be more realistic for them to act on. Therefore, an apparent limitation of Tactowel is that giving low-resolution feedback through an overall abstraction could make the performance feedback unclear. Considering this, future subtle displays should investigate how to create maximum awareness with low-resolution feedback and find the balance between them to make the feedback clear. In this direction, Khot et al. maps physical activity data to 3D printed physical forms [50]. Similar mapping approaches could be used, to give additional dimensions to the displayed performance feedback. For example, as it is proposed Tactowel changes its texture regularly. However, different areas or irregularities in the texture of Tactowel could be used to map different performance metrics.

#### 6.3.2. Overall Abstraction Could Create Ambiguity for the Players to Customize the Feedback

Alternatively, we observed that an overall abstraction creates ambiguity for the players to decide what the information is and what they should do next because it is unclear. Although we told the players Tactowel tells them if they are performing good or bad with the texture change, the players created their own motivational meaning for the feedback. These meanings were changed and customized according to participants imagining different situations. To illustrate, for receiving hard texture (You are performing bad), players thought that it means “Pull your self together!” when they are leading and “You do not have anything to lose now, attack with all you have got!” when they are loosing. Therefore, we are curious of how future subtle displays can endorse the ambiguity of the feedback to make it customizable according to the spontaneous needs of the players, such as a motivational boost.

#### 6.3.3. Tangible Interaction Adds an Affective Dimension to the Feedback to Support the Players

We learned that giving feedback through tangible interaction has several benefits to prevent distracting the players. First, the players wanted to perceive and understand the feedback instantly. In this regard, the players liked the practical and less complex approach of Tactowel. However, we can also note that the similar approach could be used by other interaction modalities, such as color change [37]. Yet, the players wanted these displays to be invisible, so they would not catch their attention along with their opponent, the referee and the audience. Thus, the second benefit is that tangible interaction, in this case texture changes, makes the feedback invisible supporting the claims of Harrison and Hudson [41]. Additionally, we discovered that choosing texture to display the feedback has a soothing or stimulating effect to boost up the motivational support. The players really liked the soft texture of Tactowel and were highly motivated to use the display. In contrast, the players shared that they feel annoyed by receiving excessive verbal feedback during the games. Unexpectedly, they associated the visual feedback from TennisKeeper with this situation and claimed that it is irritating. Therefore, we believe the third benefit is that tangible interaction can add an affective dimension to the feedback, which could support and calm the players to prevent overwhelm. On the other hand, it could also cause players to become annoyed and irritated. Previous work also supports our claim that physical properties of materials have affective dimensions, although these properties are context dependant [43]. In the light of this, we believe future subtle displays should investigate the affective impact of displaying feedback through tangible interaction and carefully design the modality to convey negative information, while still supporting the players. One way to achieve that is through psycho-physical experiments [51]. Researchers have tested their participants’ affective judgement on different physical properties of materials. Future research could utilize a similar approach for assessing their participants’ affective judgement on the presented feedback through tangible interaction.

## 7. Limitations & Future Work

The presented user study was conducted remotely during COVID-19 pandemic due to social restrictions set by Turkish Government. However, our initial aim was to conduct an user experiment in person, in which the players would play a tennis game against each other while having first hand experience with both Tactowel and TennisKeeper. The prototype of the Tactowel presented in Figure 1 was designed for this experience. It has two different textured towels that would be changed by the experimenter according to the tennis coaches’ rating of their players’ performance in a Wizard-of-Oz setting, without the knowledge of the participants [49].

We acknowledge that some aspects of the design knowledge could be lost or could not emerge in remote studies, especially for tangible interaction. As we discuss, tangible interaction utilizes the physical properties of the materials and these can induce an affective impact simply by touching the actual tangible display [19,43]. Previous research have sent these displays to their participants to acquire that impact [52]. Unfortunately, this was not possible for our case, as our Wizard-of-Oz prototype was not a stand alone product and could not be activated without the presence of a researcher. We have tried for participants to body storm with their own objects to materialize their experience, in order to negate this effect. However, motivating the participants to perform such actions was extremely challenging in the remote setting.

Still, we argue the current study yielded in adequately fruitful results to fulfil our initial aim of assessing our design strategies for Tactowel and presenting design implications for subtle displays. In this regard, we have presented five practical future directions for designing subtle displays in our discussion. We believe each one of these directions should be investigated by future research to validate and iterate the design strategies presented in this paper.

Yet, we are more intrigued in the broader design implications of subtle displays. Although we propose subtle displays for externally-paced sports, we argue the use cases could diverge to various applications. The needs of externally-paced sportspeople come from the fact that they are (1) exercising a high performance activity (physical exertion is not obligatory), (2) which demands all of their attentional resources, (3) against an opponent who changes how they will perform that activity. By applying these three requirements, we can speculate that politicians who debate against their rivals, musicians who improvise in rap battles, video-gamers who perform in real-time competitive tournaments, chess players and similar target users could also benefit from subtle displays. As our future work, we plan to conduct participatory design workshops to design a subtle display concept for one of these use cases by utilizing the design strategies presented in this paper together with their target users and interaction designers. We believe cross-referencing the findings from different use cases and design decisions would take the hypothesis of subtle displays one step further to possibly aid in creating guidelines for designing such displays across various fields.

## 8. Conclusions

In this paper, we introduce *subtle displays* that are aimed at unobtrusively displaying low-resolution real-time sports performance feedback to prevent overwhelming and distracting athletes. We apply this concept to tennis by designing *Tactowel* and evaluate it through a user study with 8 professional tennis players. Our results demonstrate three distinct design strategies and present design implications for designing subtle displays, which are the following:

(1) *Restricting the use of sports performance* displays excluding the duration of performance could prevent overwhelming and distracting tennis players. It could create an additional value of support. Other restricting strategies could be used, combined and separated. In this direction, future subtle displays should find a way to support the players, even when they are displaying a negative feedback, or they should create other restricting strategies that could block the player from trying to satisfy these displays.
(2) *Using the available routines and interactions to display sports performance feedback* could prevent overwhelming and distracting tennis players. There is coherence between using a towel and motivational feedback. Other routines and interactions could be used to attribute different meanings to the feedback. In this regard, future subtle displays should explore the coherence between different routines and their meaning for giving different kinds of feedback.
(3) *Giving an overall abstraction of sports performance through tangible interaction* could prevent overwhelming and distracting tennis players. This could create ambiguity for the players to customize the feedback and add an affective dimension to the feedback to support the players. Considering its limitations, future subtle displays should investigate how to create maximum awareness with low-resolution feedback and find the balance between them to make the feedback clear. However, future subtle displays should also endorse the ambiguity of the feedback to make it customizable according to the spontaneous needs of the players, such as a motivational boost. Additionally, future subtle displays should investigate the affective impact of displaying feedback through tangible interaction and carefully design the modality to convey negative information, while still supporting the players.

## Figures and Tables

**Table 1:** 5-point Likert scale questionnaire that communicates our design claims. Codes represent the feedback quality to be achieved and items represent the specific design goal according to that feedback quality.

#	CODE	ITEM
1	Meaningfulness	The information shared through the performance feedback would be very meaningful.
2	Importance	The information shared through the performance feedback would be very important.
3	Relevance	The information shared through the performance feedback would be highly relevant to my tennis performance.
4	Amount	The amount of information shared through the performance feedback would be adequate.
5	Resolution	The information shared through the performance feedback would be too detailed.
6	Clearness	The information shared through the performance feedback would confuse me.
7	Awareness	My awareness on my performance would increase thanks to the information shared through the performance feedback.
8	Discovery	The information shared through the performance feedback would help me to learn something about my tennis performance that I did not recognize before.
9	Reflection	The information shared through the performance feedback would lead me to think differently about my performance.
10	Perception	With this modality, the performance feedback would be very easy to perceive.
11	Understanding	With this modality, the performance feedback would be very easy to understand.
12	Reaction	With this modality, I would have a strong sense of what I am going to do, after receiving the performance feedback.
13	Cognitive Load/Overwhelm	I would invest very low mental effort on perceiving and understanding the performance feedback, and figuring out what to do, with this modality.
14	Transferability	With this modality, the performance feedback would be very coherent with the nature of a tennis performance.
15	Time	During the game, I would receive the performance feedback at the right time.
16	Intrusiveness	During the game, receiving performance feedback with this modality would not intrude my performance.
17	Distraction	With this modality, the performance feedback would not distract me from my performance.
18	Motivation	During the game, the performance feedback with this modality would increase my motivation towards the game.
19	Self-efficacy	During the game, the performance feedback with this modality would increase my belief in my own abilities.
20	Companion	During the game, the performance feedback with this modality would support me as a coach or teammate.

**Table 2:** Result of Repeated Measures *t*-test according to Likert-Scale questionnaire item pairs. Left column represents the item numbers in Table 1. Positive mean score represents TennisKeeper rated higher for the item. Note that some items, i.e., 6, are rated reverse.

#	Mean	Std. Deviation	Std. Error Mean	t	Sig(2-Tailed)	d
1	0.80000	1.30384	0.58310	1.372	0.242	1.03179
2	0.80000	1.30384	0.58310	1.372	0.242	1.03179
3	1.00000	1.22474	0.54772	1.826	0.142	1.03179
4	0.20000	1.92354	0.86023	.232	0.828	1.03179
5	0.80000	1.09545	0.48990	1.633	0.178	1.03179
6	−0.20000	0.83666	0.37417	−0.535	0.621	1.03179
7	0.80000	1.30384	0.58310	1.372	0.242	1.03179
8	1.00000	1.22474	0.54772	1.826	0.142	1.03179
9	0.20000	1.09545	0.48990	0.408	0.704	1.03179
10	−0.20000	1.48324	0.66332	−0.302	0.778	1.03179
11	0.20000	1.78885	0.80000	0.250	0.815	1.03179
12	0.60000	0.89443	0.40000	1.500	0.208	1.03179
13	0.40000	0.89443	0.40000	1.000	0.374	1.03179
14	0.60000	0.89443	0.40000	1.500	0.208	1.03179
15	0.40000	0.54772	0.24495	1.633	0.178	1.03179
16	0.20000	0.44721	0.20000	1.000	0.374	1.03179
17	−0.20000	0.83666	0.37417	−0.535	0.621	1.03179
18	0.00000	0.70711	0.31623	0.000	1.000	1.03179
19	0.80000	1.09545	0.48990	1.633	0.178	1.03179
20	0.00000	0.70711	0.31623	0.000	1.000	1.03179

## Data Availability

The data presented in this study are available on request from the corresponding author. The data are not publicly available due to participant consent form infringement.
